# Identification and management of incidental findings in a Veteran’s lung cancer screening program

**DOI:** 10.1186/s12931-025-03466-5

**Published:** 2025-12-20

**Authors:** Renee L. Colucci, Eric Garshick, Julie White, Emily S. Wan, Julianne O’Leary, Lisa A. Blake, Samantha M. Hoyt, Demerise Johnston, Kathleen LaCerda, Michelle Morreale-Karl, Anupma Jati, Fatima G. Wilder, Daniel C. Wiener, Justin M. Rucci, Ronald H. Goldstein

**Affiliations:** 1 Pulmonary, Allergy, Sleep and Critical Care Medicine Section, Medical Service, VA Boston Healthcare System, Boston, MA USA; 2 Primary Care Section, Medical Service, VA Boston Healthcare System, Boston, MA USA; 3 Radiology Section, Medical Service, VA Boston Healthcare System, Boston, MA USA; 4 Thoracic Surgery Section, Medical Service, VA Boston Healthcare System, Boston, MA USA; 5 Harvard Medical School, Boston, MA USA; 6 Brigham and Women’s Hospital Channing Division of Network Medicine, Boston, MA USA; 7 The Pulmonary Center, Department of Internal Medicine, Boston University Chobanian & Avedisian School of Medicine, Boston, MA USA

**Keywords:** Incidental findings, Lung cancer screening, Low-dose CT, Veterans, “S” modifier, Lung cancer

## Abstract

**Background:**

Incidental findings (IFs) are common on low dose CT obtained during lung cancer screening (LCS). The identification and management of clinically significant IFs is a challenging aspect of LCS programs and there is no standardized method of reporting IFs to primary care providers or patients. We explored the prevalence of incidental findings and radiologist use of the “S” modifier to identify clinically significant findings compared to what the LCS team identified as a clinically significant finding. We also presented a standardized reporting approach that provides suggested actions for providers and patients.

**Methods:**

We conducted a review of a sample of low-dose CT scans of the chest reported between August 17, 2023 and April 29, 2024 completed at VA Boston Healthcare System, omitting scans with findings concerning for lung cancer. We assessed the reporting of incidental findings by the radiologist, compared this to identification of clinically significant incidental findings by the lung cancer screening team, and evaluated factors associated with number and occurrence of incidental findings (via complete case regression).

**Results:**

Among 495 patients (Mean Age: 69.0 (6.6), 94.4% Male, 53.2% Current Smoker), 444 scans were retained for analysis. Scans had a median of seven incidental findings. The most common incidental finding was multiple pulmonary nodules (77.9%). There were 165 low-dose CT with findings the lung cancer screening team considered clinically important, however, radiologists only assigned the “S” modifier to 35 scans.

**Conclusions:**

All scans reviewed had incidental findings and nearly 40% had a finding that was clinically significant to the lung cancer screening team. There was inconsistent reporting between the radiologists and lung cancer screening team on clinical significance. Radiologists identified fewer clinically important findings than the lung cancer screening team and applied the “S” modifier inconsistently. There is no standardized method for reporting clinically significant low-dose CT results to primary care providers or patients. Our approach provides a structured approach, acknowledges multiple clinical opinions, and provides a framework for communication.

**Supplementary Information:**

The online version contains supplementary material available at 10.1186/s12931-025-03466-5.

## Introduction

Lung cancer screening (LCS) attempts to identify treatable early lung cancers in the form of solitary pulmonary nodules. The US Preventive Services Task Force (USPSTF) recommends annual LCS by low-dose CT scan (LDCT) for adults aged 50 to 80 years who have at least a 20 pack-year smoking history and are either current smokers or have quit within the last 15 years.[1]During this process, other radiologic abnormalities detected by CT scanning are considered incidental findings (IFs).[2] Reported prevalence of IFs identified during LCS varies widely (7.5−90%) and includes IFs of varying significance without determination of clinical significance or need for further actions by the LCS team or referring providers.[3–7] Common IFs include emphysema, coronary artery calcifications (CAC), multiple pulmonary nodules, and interstitial lung abnormalities.[8, 9].

The Lung CT Screening Reporting & Data Systems (Lung-RADS^®^) version 2022 advocates for standardized reporting of pulmonary nodules and IFs in CT reports.[10]For IFs, the radiologist may use the “S” modifier if there is a “new or changing clinically significant or potentially clinically significant finding unrelated to cancer.”[11]The v2022 description of the “S” modifier clarified the previous definition (v1.1, 2019), however, there is still variability between and within lung cancer screening programs on which findings warrant the “S” modifier – particularly in populations, such as Veterans, where findings such as interstitial abnormalities or emphysema may be clinically relevant but not unexpected.[4, 7, 10, 12–16].

Incidental findings may be clinically relevant and require attention by non-pulmonary providers, including the patient’s primary care physician. Inadequate communication of these findings among providers can be problematic, for example, leading to delayed diagnoses, particularly when additional procedures are required as a next step in evaluation.[5–7, 17] An additional issue relates to the communication of LDCT results, including IFs, to patients as required by the Department of Veterans Affairs (VA) regulations. The VA Boston LCS program attempted to address this issue by developing descriptive information for patients and thereby serving to reduce the burden to the primary care providers.

A multidisciplinary team requires effective communication and management plans to report IFs.[18]Pulmonologists, radiologists, and primary care physicians agreed that an important factor to LCS program success is creating and maintaining a supported, centralized program.[19] To effectively communicate IFs at our Veteran’s healthcare system, the LCS team collaborated with the primary care service to develop institutional guidelines regarding the clinical significance of IFs and methods of reporting IFs to primary care providers and patients.

In this study, we performed a retrospective chart review of LDCT conducted in an established LCS program at the VA Boston Healthcare System (BHS) over a six-month period. We applied our institutional guidelines to findings that required notification of primary care providers (PCPs) and if further treatment, procedures, or review were required within one month. We sought to identify common IFs, their frequency and clinical significance, and describe a framework for effective communication of LCS findings with primary care physicians and patients.

## Methods

### Overview of the VA Boston lung cancer program approach to incidental findings

VA BHS consists of three hospitals and four community-based clinics. It utilizes a centralized LCS program, consisting of a nurse practitioner, several registered nurses, and a pulmonary physician, all of whom are dedicated to the LCS program. Patients referred by primary care are confirmed eligible by the LCS nurses, who order LDCT as necessary. Radiologists with specialized training in LCS LDCT serve as the primary readers of the LDCT.

Following each patient’s LDCT, VA BHS radiologists are expected to use Lung-RADS^®^ v2022 guidelines to assign Lung-RADS^®^ scores and identify IFs within LDCT reports. The Lung-RADS^®^“S” modifier is recommended for any scan with clinically significant findings unrelated to lung cancer.[11] The radiologists use a standardized template to ensure consistent reporting of findings provided by the National Center for Lung Cancer Screening and report any incidental finding identified on the LDCT, regardless of newness or stability.

The LCS team reviews all LCS radiological reports and enters a note into the electronic medical record (EMR) that includes the Lung-RADS^®^ designation, a list of any IFs, and recommendations for follow up. The team compares these reports to previous LDCT reports (if available) to determine if the finding is new, worsening, or previously reported. All Lung-RADS^®^ 0, 3 or 4 are reviewed with the VA Boston LCS NP or pulmonologist. As indicated for complex problems, the abnormal scans are reviewed at a multidisciplinary conference, including the pulmonary, thoracic surgery, and radiology departments. All reviews and recommendations are noted in the chart.

In the hopes of improving follow up of IFs detected on LDCT, the LCS team and primary care department collaborated to (1) categorize IFs by clinical significance, (2) develop an approach to communicate IFs based on clinical significance, and (3) communicate details about IFs to patients.

IFs were divided into three categories (Table 1), with categories assigned independently to each finding. The first category, actionable findings, involves clinically significant findings that require additional testing, treatment, or review within one month of discovery and are determined by the LCS team with occasional input from the radiologist. These exclude new or enlarging lung nodules suspicious of lung cancer that are not incidental. Some IFs were only considered actionable in certain instances, such as severe CAC only being actionable if a specialty referral was required. For IFs that are actionable, the PCP receives direct notification from the LCS nurses (by encrypted electronic messaging or telephone) and require primary care co-signature on the report. The results are communicated to the patient via telephone call or certified letter (if unable to reach patient via telephone).

**Table 1:** Lung cancer screening and primary care defined levels of clinical significance for LDCT findings

Level of Clinical Significance	Findings Identified by Primary Care and LCS Teams
Clinically Significant, Actionable *– Requiring further imaging/testing or treatment within one month of scan report. Primary Care notified by telephone or encrypted electronic message.*	• Coronary artery calcifications^a^
	• Enlarging lymph nodes
	• Pericardial effusion
	• Pleural effusion
	• Pneumonia
Clinically Significant, No Follow Up Needed Within 30 Days – *Primary Care is notified through request of electronic co-signature on chart note describing findings in the patient’s medical record.*	• Aortic Aneurysm > 4.5 cm
	• Asymmetrical gynecomastia^b^
	• Bronchial wall thickening
	• Bronchiectasis
	• Cirrhosis
	• Esophageal thickening
	• New or worsening COPD/Emphysema
	• Pulmonary fibrosis
	• Pleural plaques
	• Thoracic Aneurysm > 4 cm
Not Clinically Significant – *LCS Team places chart note in the patient’s medical record*,* but Primary Care is not immediately alerted. Findings may be discussed with patient at next Primary Care appointment.*	• Atelectasis
	• Atherosclerosis
	• Bi-apical scarring
	• Bone spurs/Osteophytes
	• Calcified granulomas
	• Cholelithiasis
	• Degenerative disc disease
	• Diverticulosis
	• Fatty liver
	• Gallstones
	• Gastroesophageal Reflux Disease (GERD)^c^
	• Gynecomastia
	• Hiatal hernia
	• Kyphosis

^a^If referral to other medical specialty is required

^b^If mammogram is recommended

^c^Patients were considered to have GERD if they were on a reflux medication or had a diagnosis of GERD in their medical chart

The second category consists of clinically significant findings that do not require immediate attention but warrant primary care awareness. These findings (Table 1) require a primary care electronic co-signer on the LCS results note in the patient’s record but no direct outreach to the PCP. The third group consists of findings that are deemed clinically insignificant, do not require primary care co-signature, and if appropriate, can be discussed at the patient’s next PCP visit. Patients without an actionable finding receive a letter containing a copy of the radiological report, and a standardized definition of any IF listed in the report, written at an 8th grade level (Additional Table 1).

### Setting and cohort

To assess prevalence and management of IFs within our LCS program, we reviewed a sample of 500 reports from LDCTs, conducted between August 17, 2023, and April 29, 2024. VA BHS radiologists assigned Lung-RADS^®^ scores using the Lung-RADS^®^ v2022 guidelines and identified IFs on the LDCT. For analysis, we omitted any LDCT reports with Lung-RADS^®^ 3 or 4 classification or that identified a pulmonary nodule otherwise suspicious for lung cancer (but did not receive a Lung-RADS^®^ 3 or 4) that required further diagnostic work-up. The radiologist assigned the Lung-RADS^®^ “S” modifier to any scan they determined had a clinically significant finding unrelated to lung cancer. Lung-RADS^®^ scores and findings were input as a radiology report in the patient’s EMR. For any patient with more than one LDCT in the study period, we retained the earliest completed LDCT for analysis.

LCS nurses selected the first 5 to 10 CT reports of each day during the study period to be systematically reviewed for IFs and to extract pertinent patient data from the patient’s EMR. We identified patient demographics (age, sex, body mass index [BMI], race/ethnicity), smoking status and cigarette pack-year history at time of LCS consult. All incidental findings – as defined by the LCS and primary care collaboration – that were mentioned in the radiology report were recorded, including both pulmonary and non-pulmonary IFs (Additional Table 2). When nurses identified an IF in a radiology report that did not align with pre-defined IF categories, they recorded the finding via free text. Pulmonologists and pulmonary nurse practitioners reviewed these IFs and assigned them to the appropriate category. Lung-RADS^®^ “S” modifiers assigned by the radiology service were also recorded.

### Statistical analysis

Demographic and smoking-related variables were summarized using means (standard deviations) or counts (percentages) as appropriate. We describe rates of IF detection overall and stratified by pulmonary and non-pulmonary findings. Multivariable regression using complete case analysis was used to model the possible effects of the demographics and outcomes including total IFs per LDCT (linear regression) and presence of an actionable finding (logistic regression).

All analyses and figures were generated using SAS software and R Studio.[20–24] All study activities were approved by the VA Boston Institutional Review Board with a waiver for informed consent.

## Results

Between 8/17/2023 and 4/29/2024, 1,092 LDCT scans were performed at VA BHS. 500 reports from 495 unique patients were selected for review. Certain reports (51) were excluded due to the presence of a finding suspicious for cancer, resulting in 444 reports retained for analysis. The mean (SD) age and years enrolled in LCS at time of LDCT were 69.0 (6.6) and 4.2 (2.1) years, respectively (Table 2). Patients were primarily male (94.4%), non-Hispanic (98.8%), and White (87.0%). 75% of patients were overweight or obese. Over half (53.2%) of patients were current smokers at the time of the selected LDCT and had an average of 46.9 (24.4) pack-years at their initial LCS consultation.

**Table 2:** Cohort demographics

		Determined Clinically Significant by LCS Team		
	Unique Patients^a^ *N* = 444	Actionable Finding, Incidental *N* = 32	Clinically Significant, Not Actionable *N* = 133	No Clinically Significant Finding *N* = 279
Age, Mean (SD), Years	69.0 (6.6)	69.8 (7.3)	69.4 (6.6)	68.7 (6.6)
Years Enrolled in LCS, Mean (SD)	4.2 (2.1)	3.8 (2.1)	4.0 (2.2)	4.3 (2.0)
BMI^b^ *(n=441)*				
Underweight or Normal Weight	110 (24.5)	13 (40.6)	35 (26.7)	62 (22.3)
Overweight	161 (36.5)	7 (21.9)	46 (35.1)	108 (38.9)
Obese	170 (38.6)	12 (37.5)	50 (38.2)	108 (38.9)
Gender				
Male	419 (94.4)	30 (93.8)	130 (97.7)	259 (92.8)
Female	25 (5.6)	2 (6.3)	3 (2.3)	20 (7.2)
Ethnicity *(n=430)*				
Hispanic or Latino	5 (1.2)	0 (0.0)	1 (0.8)	4 (1.5)
Not Hispanic or Latino	435 (98.8)	32 (100.0)	128 (99.2)	265 (98.5)
Race^c^ *(n=431)*				
White	375 (87.0)	29 (93.6)	108 (84.4)	238 (87.5)
Non-White	56 (13.0)	2 (6.4)	20 (15.6)	34 (12.5)
Current Smoker at Time of LDCT				
Yes	236 (53.2)	14 (43.8)	76 (57.1)	146 (52.3)
No	208 (46.8)	18 (56.2)	57 (42.9)	133 (47.7)
Pack Years at Initial Consult *(n=411)*	46.9 (24.4)	56.1 (34.2)	46.1 (28.1)	46.1 (20.7)
RADS Score^d^				
0	15 (3.4)	13 (41.9)	0 (0.0)	2 (0.7)
1	28 (6.3)	1 (3.2)	5 (3.8)	22 (7.9)
2	400 (90.3)	17 (57.8)	128 (96.2)	255 (91.4)
S Modifier Assigned by Radiologist^c^				
Yes	35 (7.9)	10 (31.3)	14 (10.5)	3 (1.1)
No	409 (92.1)	22 (68.8)	119 (89.5)	276 (98.9)

^a^Continuous variables are presented as Mean (Standard Deviation); Categorical variables are presented as N (%)

^b^Numeric BMI is categorized based on CDC recommendations for adults: Underweight or Normal Weight (less than 25.0), Overweight (25 to less than 30), and Obese (30 or greater). 4 patients were considered underweight (BMI < 18.5)

^c^‘Non-White’ race includes: African American or Black (*n*=48), American Indian or Alaska Native (*n*=5), Asian (*n*=2), and Native Hawaiian or Pacific Islander (*n*=1)

^d^One scan did not have a RADS score assigned

### Prevalence of incidental findings

Incidental findings were highly common, as all 444 LDCT reviewed had at least one IF reported. Common IFs were representative of both pulmonary (Table 3) and non-pulmonary (Table 4) findings. Most LDCT reports (95.5%) had at least one each pulmonary and non-pulmonary finding, although non-pulmonary IFs were identified on more scans than pulmonary IFs (437, 98.4% and 431, 97.1%, respectively). Presence of multiple nodules, skeletal abnormalities, coronary artery calcifications, and emphysema were all identified on at least 50% of LDCT reports in this sample.

**Table 3:** Frequency of pulmonary findings identified on LDCT

	Number of Scans (*N* = 444)
Presence of Multiple Nodules	346 (77.9)
Emphysema	269 (60.6)
Scarring	127 (28.6)
Granulomas	65 (14.6)
Atelectasis	54 (12.2)
Pulmonary Infiltrates	29 (6.5)
Interstitial Changes ^a^	23 (5.2)
Bronchiectasis	22 (5.0)
Airway-related Finding ^b^	17 (3.8)
Pleural Plaques	17 (3.8)
Pulmonary Vascular *(ex. Pulmonary Artery Enlargement)*	10 (2.3)
Pulmonary Lymph Nodes	8 (1.8)
Diaphragm-Related Finding ^c^	5 (1.1)
Azygos Lobe	4 (0.9)
Pleural Effusion	3 (0.7)
Hamartoma	1 (0.2)

^a^Included but not limited to: Interstitial Changes, Reticular Changes, Interstitial Pulmonary Fibrosis

^b^Included but not limited to: Bronchial Wall Thickening, Diverticulum

^c^Included but not limited to: Elevation of Hemidiaphragm

**Table 4:** Frequency of non-Pulmonary findings identified on LDCT

	Number of Scans (N = 444)
Miscellaneous Skeletal Abnormalities ^a^	316 (71.2)
Degenerative Changes of the Spine	296 (93.7)
Old/Healed Fractures	14 (4.4)
Scoliosis	6 (1.9)
Bone Spur/Osteophytes	1 (0.3)
New Fractures	1 (0.3)
Kyphosis	1 (0.3)
Coronary Artery Calcifications	297 (66.9)
Other Calcifications	218 (49.1)
Gastroesophageal Reflux Disease (GERD)	193 (43.5)
Other GI Finding ^a^	147 (33.1)
Cholelithiasis	30 (6.8)
Diverticulosis	22 (5.0)
Liver Lesions	19 (4.3)
Hepatic Steatosis	18 (4.1)
Liver Cyst	13 (2.9)
Spleen Abnormalities	14 (3.2)
Adrenal Adenoma	10 (2.3)
Renal	77 (17.3)
Atherosclerosis	66 (14.9)
Vascular Finding (ex. Aneurysm)	49 (11.0)
Gynecomastia	44 (9.9)
Esophageal Finding or Hiatal Hernia ^a^	35 (7.9)
Esophageal Thickening	3 (0.7)
Patulous Esophagus	2 (0.5)
Cardiac Finding (ex. Cardiomegaly, Pericardial Effusion)	22 (5.0)
Thyroid	11 (2.5)
Aorta-Related Finding	8 (1.8)
Abnormalities of Skin or Chest Wall ^b^	7 (1.6)
Cirrhosis	2 (0.5)

^a^Subcategories may not represent all findings within the category

^b^Included but not limited to: Lesions, Soft Tissue Attenuation of Chest, Subcutaneous Nodule of Chest Wall

**Fig. 1:**
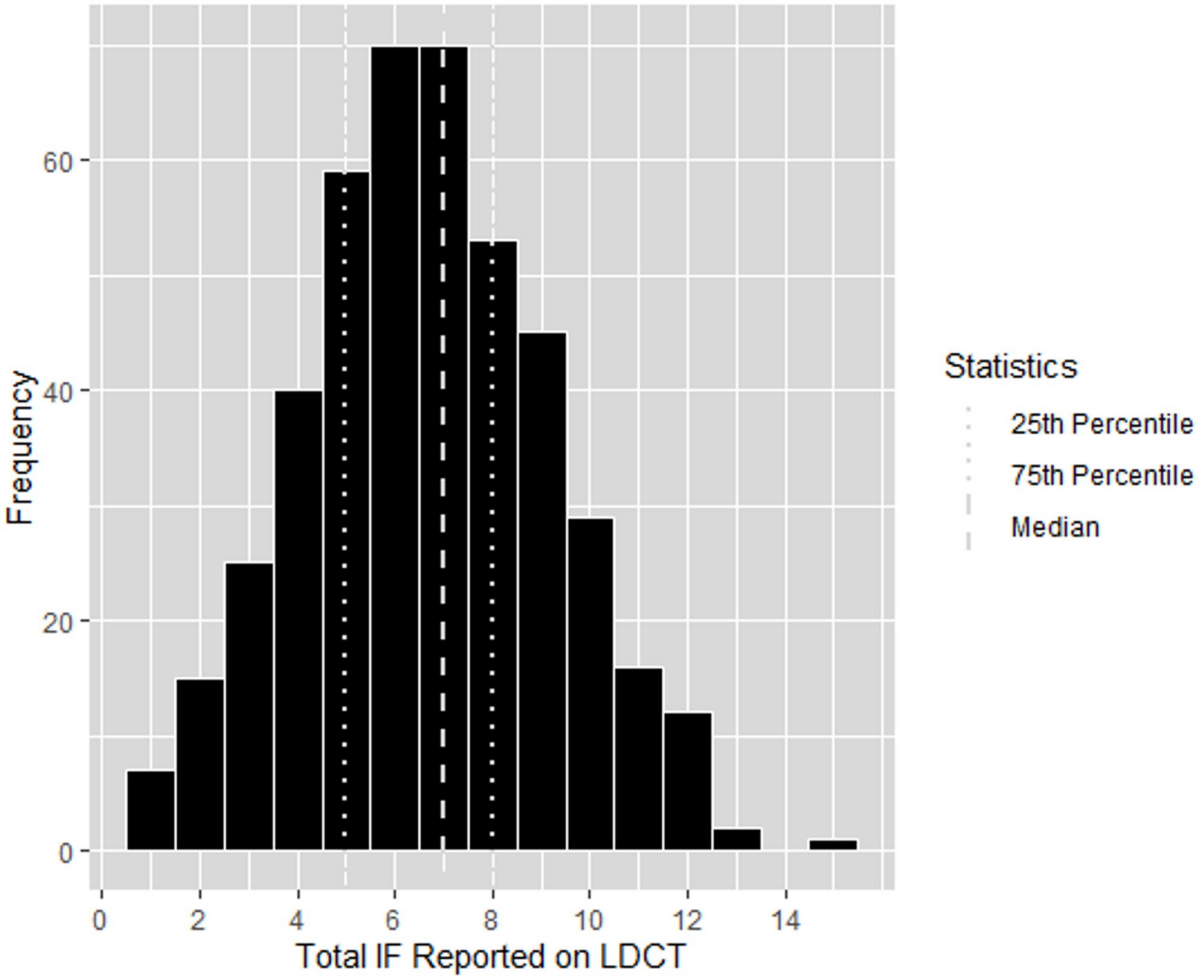
Number of incidental findings reported per LDCT. Median: 7, 25th Percentile: 5, 75th Percentile: 8

Each LDCT had a median of 7 IFs (Range: 1–15) identified in the report (Fig. 1). Univariable and multivariable analyses are summarized in Additional Table 3. In a multivariable model, age and smoking status at the time of the LDCT were significantly associated with changes in the total number of IFs reported. A 10-year increase in age was associated with an increase in nearly one IF increase per scan (0.97 [95% CI: 0.59, 1.4]), whereas current smokers had on average, 0.5 fewer IFs than former or non-smokers (95% CI: −1.0, −0.03).

### LCS team identification of clinically significant incidental findings

The LCS team identified at least one IF considered clinically significant on 165 (37.2%) reports, which required PCP co-signature on the report note in the patient’s medical chart. A chart review found that PCP signed 154 of 159 (96.9%) notes the LCS nurses requested a signature on. Six scans did not have a PCP signature requested. In 5 of these cases, the clinically significant finding was seen on a prior LDCT scan and previously reported to the PCP. We identified only 1 of 165 scans in which our LCS team should have requested a PCP co-signature but did not. Thirty-two (19.4%) reports with a clinically significant finding required PCP alert and warranted additional review or treatment within 30 days of discovery (defined as an ‘actionable’ finding). In a multivariable model, a 10-year increase in age was associated with a 77% (3%, 205%) increase in odds of having an actionable IF (Additional Table 4). Additionally, overweight patients, compared to underweight or normal weight patients, had a 59% decrease in odds of having an actionable IF (Additional Table 4).

### Pulmonary incidental findings

There were 431 (97.1%) LDCT reporting at least one pulmonary IF. Multiple pulmonary nodules (77.9%), emphysema (60.6%), and scarring (28.6%) were the three most common pulmonary IFs identified. There were a variety of pulmonary findings reported; in total, 16 unique pulmonary IFs were identified across all scans reviewed.

The LCS team followed 23 actionable IFs, all of which were reviewed with the LCS pulmonologist. The patient’s PCP signed the report for each of these findings. Nearly all (21, 92.3%) of these findings were related to opacifications or consolidations and the remaining two actionable findings were pleural effusions. Actions taken in response to these findings included a clinic visit (8, 34.8%), antibiotics (3, 13.0%), or a bronchoscopy (1, 4.4%). Additionally, 17 (73.9%) of the findings required accelerated radiologic follow up.

### Non-Pulmonary incidental findings

Non-pulmonary IFs were identified on 437 (98.4%) LDCT reviewed. Miscellaneous skeletal abnormalities were the most common non-pulmonary IF (316 LDCT), of which, degenerative changes of the spines were identified on 296 (93.7%) of reports with skeletal IFs (Table 3). Calcifications were also common, as 66.9% and 49.1% of LDCT had CAC or other calcifications reported, respectively. Fifteen different non-pulmonary IFs were identified in our sample.

Nine actionable findings were followed by the patient’s PCP. Five of these findings were severe CAC, two findings were related to the aorta (aortic pseudoaneurysm and ectatic aorta), and one each thyroid nodule and soft tissue mass in the abdomen. PCP co-signature was requested on all nine reports containing these actionable findings and was obtained on eight (88.9%). These findings were either discussed at the patient’s next PCP appointment if the finding was stable or the PCP initiated a referral to the appropriate specialty.

### Radiologist identification of clinically significant findings (“S” Modifier)

The Lung-RADS^®^“S” Modifier is used by the radiologist to describe IFs defined as “clinically significant or potentially clinically significant findings unrelated to lung cancer.”[11] There were discrepancies between the clinically significant findings identified by the LCS team as compared with the radiological determination using the “S” modifier. Thirty-five (7.9%) LDCT reports were assigned the “S” modifier. Of the 165 reports with a finding clinically significant to the LCS team, 31 (18.8%) reports also had an “S” modifier assigned.

The LCS team identified 23 reports with pulmonary actionable findings. The reading radiologist co-identified only 3 of these reports with the “S” modifier (one pleural effusion and two instances of opacities). Thirteen of these reports were designated as Lung-RADS^®^0, suggestive of an inflammatory or infectious process but not requiring the “S” modifier.[11].

There was inter- and intra-radiologist variability in the reporting of the “S” modifier. One radiologist who identified severe CAC on three reports only categorized it using the “S” modifier on two reports. Similarly, pleural effusion was identified on three reports, however, only one report received the “S” modifier. The radiologist who assigned the “S” modifier identified a pleural effusion on a second report as well but did not assign the “S” modifier.

## Discussion

In a review of 444 LDCT in a Veteran population, IFs were identified on every scan, with a median of seven IF reported per scan. Multiple nodules, emphysema, skeletal abnormalities, and coronary artery calcifications were the most common findings – all found on more than half of the scans reviewed. Only a minority of these findings also received an “S” modifier on the radiology report.

The organization of lung cancer screening programs varies among healthcare systems. Centralized centers, such as VA BHS, have a dedicated team to manage LCS findings whereas decentralized centers lack an LCS specific team. Multiple departments are involved in LCS and miscommunication between primary care and LCS can potentially result in missteps surrounding patient care.[17]As seen in our study, IFs are ubiquitous on LDCT and can have varying levels of clinical significance. It is critical that key members of the patient’s care team, including their primary care team and LCS coordinators, have defined and agreed upon roles to ensure effective care. Our LCS team has operationalized this approach by collaborating with primary care to establish a practical process for notifying PCPs about IFs. This allowed each department to identify what is clinically important to them and to design different reporting pathways depending on the clinical significance of IFs. Our multidisciplinary approach emphasizes PCP notification for high-acuity IFs and has the potential to reduce feelings of burnout related to over-notification of clinically insignificant IFs. Previous studies described the need to report IFs to referring providers but have not prioritized the significance of the IF.[25, 26] We found that a limited number of IFs (7.2% [32]) were actionable and even fewer required primary care follow up (2% [9]). These results suggest that the burden to PCPs can be minimized by an effective organizational structure.

Appropriate communication of LDCT results with patients can ensure patient and provider satisfaction. A previous evaluation of LCS programs across VA hospitals described different methods of communicating screening results, including mailing letters or pamphlets or verbal disclosure in-person or by telephone.[27]Patients who received letters (usually those with normal or low-risk findings) expressed frustration by medical language used to describe findings.[27]Physicians noted that they understood the confusion patients may face when receiving these letters, but ultimately waited for the patients to reach out with questions or concerns.[27].

The VA Boston LCS team developed a process for communicating incidental findings to the patient with the goal of improving patient understanding of these findings. The team uses a letter template that includes the “findings” section of the radiologist report, the general definition of IF, and a standardized definition of any IFs listed in the report, written at an 8th grade level. The letter instructs patients to call the LCS team nurses for any questions regarding follow-up of pulmonary nodules or to contact their PCP for questions related to other findings. The benefits of these letters, while not explicitly studied in this project, can serve as a next step in determining the effectiveness of our methodology.

Only a minority of IFs deemed clinically significant by the LCS and PCP team had an “S” modifier assigned to them within the radiologists’ report and there was inter- and intra-radiologist variability in use of “S” modifiers. Lung-RADS^®^0 assignment, in some cases, further complicates the process, as this designation usually implies an actionable IF. These discrepancies echo previously identified challenges related to variable application ”S” modifiers in the real-world and in defining clinically significant IFs.[7, 15, 16] Lung-RADS^®^criteria do not require an “S” modifier for findings that are clinically stable, which may account for some of the discrepancies in identification of clinically significant findings. Refining a list of clinically significant IFs to provide to the radiology service may reduce the potential that these findings go untreated or are over reported. IF management has been identified as a barrier to LCS implementation, so any method to [1]standardize the reporting and management of IFs or [2]delineate clear responsibilities for follow up might alleviate challenges some LCS programs face when starting or enlarging a screening program.[28] Within the VA healthcare system, the National Center for Lung Cancer Screening conducts a monthly meeting providing an opportunity to disseminate new information, guidelines, and frameworks to other programs.

Lung cancer screening cannot be considered a linear process, as participants may be referred to the program by their PCP, only to potentially be sent back for management of IFs. PCPs in de-centralized systems have expressed concerns that follow up of LCS findings can be difficult to manage within the host of other medical issues they are tasked with addressing.[19]A benefit of the centralized method of LCS reporting at VA BHS is the efficient means of communication with primary care to ensure timely follow up for findings that require it. Previous meta-analyses have found that LCS participant adherence to annual screening after negative baseline LDCT is higher in centralized LCS programs than de-centralized and our work suggests there may be similar benefits to reporting and follow up of IFs in centralized programs.[29].

### Limitations

This study had several limitations. First, it was conducted in a single Veterans Healthcare System with a highly resourced, centralized LCS program with few women and non-white LCS participants. In this population, the potential for various military exposures may limit generalizability to other settings or impact the rate of IF detection. This information may also not be generalizable to patients who receive their care within multiple healthcare systems, instead of a more centralized system within the VA. Additionally, although a longitudinal process, our study only accounted for one LDCT per person, which may not have always accurately represented the newness or stability of the IFs reported.

## Conclusion

Many studies and analyses have shown the frequency of IFs, yet there have been significantly fewer studies about communication and management of these findings. Notably, the prevalence of IFs requiring follow up action was not previously addressed. Lung cancer screening can become complex when considering the number of involved (both patients and physicians). VA BHS has developed a plan to communicate IFs between the primary care and pulmonary departments, as well as providing patient education. This framework and collaborative approach can be introduced and tailored to other healthcare system’s needs, promoting an effective approach to management of IFs detected within an LCS program.

## Supplementary Information

Supplementary Material 1. Additional Table 1. Standardized Definitions of Incidental Findings Used for Patient Education (Colucci_et_al_AddFile1.docx)

Supplementary Material 2. Additional Table 2. Categories of Incidental Findings Reviewed on LDCT (Colucci_et_al_AddFile2.docx)

Supplementary Material 3. Additional Table 3. Demographics and Potential Relationship to Total Incidental Findings per LDCT (Colucci_et_al_AddFile3.docx)

Supplementary Material 4. Additional Table 4. Demographics and Potential Associations to Presence of an Actionable Incidental Finding on a LDCT (Colucci_et_al_AddFile4.docx)

## Data Availability

The data that supports the findings of this study are not openly available due to patient privacy and sensitive data, but a de-identified version of the data may be available upon reasonable request. All requests for data must be made in writing to the corresponding author.

## Abbreviations

LCS: Lung Cancer Screening
IF: Incidental Finding
CAC: Coronary Artery Calcifications
Lung-RADS®: Lung CT Screening Reporting & Data Systems
VA: Department of Veterans Affairs
LDCT: Low-dose CT Scans
BHS: Boston Healthcare System
PCP: Primary Care Provider
LPOP: Lung Precision Oncology Program
EMR: Electronic Medical Record
BMI: Body Mass Index
