# Supplementary material for: Identification and management of incidental findings in a Veteran’s lung cancer screening program
Source: Respir Res. 2025 Dec 20;27:24. doi: 10.1186/s12931-025-03466-5 (PMC12836813; doi:10.1186/s12931-025-03466-5)
Supplement: Supplementary file 1 — Supplementary Material 1. Additional Table 1. Standardized Definitions of Incidental Findings Used for Patient Education (Colucci_et_al_AddFile1.docx) [file 12931_2025_3466_MOESM1_ESM.docx]

**Additional Table 1. Standardized Definitions of Incidental Findings Used for Patient Education**

| **Incidental Finding** | **Definition** |
| --- | --- |
| Abdominal Aortic Aneurysm (AAA) | **Abdominal Aortic Aneurysm (AAA)** is a problem in the aorta, the main blood vessel that comes out of the heart. Blood flows through the aorta to the rest of the body. The abdominal aorta is the part of the aorta that is located in the stomach area. It branches to bring blood to the organs in the stomach area and the legs. In people with an **AAA**, a part of the abdominal aorta balloons out or bulges. If the bulge bursts, the condition becomes very dangerous. A burst aorta causes lots of internal bleeding. Your provider will discuss your need for further testing and/or treatment. |
| Atelectasis | **Atelectasis** is the collapse of one or more sections (lobes) of the lungs. When you breathe in, the lungs normally expand to fill with air. With atelectasis, a blockage or pressure in the area around the lung (pleura)keeps the lung from expanding. |
| Atherosclerosis | **Atherosclerosis** is when your arteries narrow and harden. This occurs **over many years**. Fat and cholesterol start to build up on your artery walls. This is also called coronary artery disease. This **may** restrict blood flow and causes clots to form. Atherosclerosis does not produce symptoms until an artery becomes narrow or blocked. Your provider will discuss your need for further testing and/or treatment. |
| Biapical scarring | **Biapical scarring** is scar tissue at the top of both lungs (**apical** - the top of the lung, **base** - the bottom of the lung). |
| Bone Spur or Osteophyte | **Bone Spur or Osteophyte** is a tiny and pointed outgrowth of bone. Bone spurs occur when your body adds more bone tissue to fix a damaged bone. It can happen at the site of a joint or tendon injury and can be seen in arthritis (osteoarthritis) or tendonitis. This is generally not a concerning finding. |
| Bronchial Wall Thickening | **Bronchial Wall Thickening** is caused by inflammation and infections. This may result in extra mucus production. Thicker walls keep mucus from draining the way it normally should. The extra mucus can trap bacteria, causing more infections, scarring, and thickening. |
| Bronchiectasis | **Bronchiectasis**- when the airways (bronchi) in the lungs are damaged, widened, and sometimes thickened. This condition makes it hard for the lungs to get rid of mucus, and it gathers in the bronchi. This may lead to lung infections, which can make the condition worse. It is very important that you bring up the mucus. |
| Calcified Granuloma | **Calcified Granuloma** comes from old (healed) inflammation in body tissue. The inflammation that collects calcium during the healing process. This is usually a type of scar from an old infection or injury. |
| Cholelithiasis | **Cholelithiasis** happens when gallstones form in the gallbladder (an organ near your liver where bile is stored). Bile is a fluid that helps digest fats and can harden and form into gallstones. Gallstones can cause painful blockages. This is often referred to as a gallbladder attack. |
| Cirrhosis | **Cirrhosis** results from long-term (chronic) damage to your liver. The liver turns food into energy and helps keep your blood free from toxins. It makes important proteins and absorbs needed vitamins from food. Your provider will discuss your need for further testing and/or treatment. |
| Coronary Artery Calcification | **Coronary Artery Calcification** happens when calcium builds up in the vessels that provide blood and oxygen to your heart. Too much calcium causes the artery walls to become hardened. This may reduce the amount of oxygen to your heart. |
| Degenerative Changes in the Spine | **Degenerative Changes in the Spine** are a form of arthritis that weakens your bones over time. This is called osteoarthritis. It is most often found in the neck and lower back. Doctors may also call this finding a degenerative arthritis or degenerative joint disease. |
| Diverticulosis | **Diverticulosis** is a condition when small pouches (diverticula) form in your colon (large intestine) wall. The colon is where water is absorbed, and stool is formed. The pouches form in the colon when the inside layer pushes through weak spots in the outer layers. You may have a few pouches or many of them. This may be due to constipation (having hard stools, straining, or less than three bowel movements per week). Constipation occurs when there is not enough fiber in the diet, or other causes. |
| Emphysema | **Emphysema** is a type of damage to the small air sacs (alveoli) in the lungs. The damage to the small air sacs looks like holes in the lung on a CT scan. Normal air sacs carry oxygen to your blood when you breathe. Damaged air sacs (emphysema) in some cases carry less oxygen to your blood. Emphysema can be seen with chronic obstructive pulmonary disease (COPD), which can also make breathing more difficult. |
| Fatty Liver Disease | **Fatty Liver Disease** is when you have a buildup of extra fat in your liver. The fat deposits **may** cause liver damage. Fatty liver disease is also called hepatic steatosis or steatohepatitis. Your liver helps keep your blood free from toxins and produces fluids that your body needs. It helps your body use and store energy from the food you eat. Your provider will discuss this with you. |
| Fibrosis or Fibrotic Change | **Fibrosis or Fibrotic Change** is scarring around the tiny air sacs (alveoli) in your lungs. Fibrosis causes the tissue in the lungs to get thick and stiff. This makes it hard to get oxygen into your blood. Mild fibrosis may not cause any problems, but a lot of fibrosis may cause shortness of breath or low oxygen levels. Your provider will discuss your need for further testing and/or treatment. |
| Gallstones | **Gallstones** are located in your gallbladder. Your gallbladder stores bile, a fluid made by the liver. Bile helps digest fats in the foods you eat. When certain substances in the bile change and become solid, they are called gallstones. If gallstones block the flow of bile, it may cause pain or infection known as a gallbladder attack. However, many people with gallstones may not have gallbladder attacks. |
| Gastroesophageal Reflux Disease | **Gastroesophageal Reflux Disease** is when acid and food in your stomach flows back through a tube (esophagus) that connects to your mouth. Reflux can happen if you drink caffeinated beverages, smoke, or have big or spicy meals. Your provider will contact you to discuss GER. |
| Ground Glass Opacity (GGO) | **Ground Glass Opacity (GGO)** refers to hazy gray areas (like “frosted glass”) in the lungs. Choking (on food, liquid, or body secretions), infections, inflammation, fluid, or abnormal growths can cause GGO. Your provider will check for possible causes and discuss your need for further testing and/or treatment. |
| Gynecomastia | **Gynecomastia** is when male breast tissue swells and gets bigger. This can be caused by certain medications, changes in hormones, and being overweight. You can discuss this with your provider. |
| Hiatal Hernia | **Hiatal Hernia** happens when the stomach slides above the diaphragm. The diaphragm is a muscle that separates your stomach from your chest. A hiatal hernia can occur at birth or over time. |
| Kyphosis | **Kyphosis** is an abnormal curving of the upper back. Bones in your upper back become wedge-shaped. This causes a rounded or hunched back that might hurt or feel stiff. Osteoporosis, degenerative disc disease and weak lower back muscles may case kyphosis. Kyphosis is sometimes called dowager hump. It is most common among elderly people, but it can occur at any age. |
| Pericardial Effusion | **Pericardial Effusion** is when your heart has too much fluid around it. The pericardium is a double-layered, saclike structure around your heart. This structure contains a thin layer of fluid. When the pericardium is weak from disease or injury, this causes inflammation and excess fluid to build up. If this is a new finding for you, your provider will discuss your need for further testing and/or treatment. |
| Pleural Effusion | **Pleural Effusion** is a fluid build-up in the space between your lungs and the wall of your chest. Normally, there is a small amount of fluid in this space. Some conditions can cause a large amount of fluid to build up. A large amount of fluid in this space can cause the lung to collapse if not treated. Your provider will discuss your need for further testing and/or treatment. |
| Pleural Plaques | **Pleural Plaques** are areas of benign thickening in the lining of your lungs. They usually do not require treatment. Plural plaques develop after extended exposure to asbestos. Most patients with pleural plaques do not experience a loss of lung function or have any symptoms. |
| Pneumonia | **Pneumonia** is an infection in your lungs. Inside the lungs are tiny air sacs (alveoli). These air sacs help get oxygen into your bloodstream when you breathe. Pneumonia causes swelling and irritation of these air sacs. The air sacs may fill with fluid or pus and cause you to cough or have trouble breathing. There may be less oxygen in your blood to make your body work properly. Pneumonias can be caused by viruses, bacteria, or other organisms. Your provider will discuss your need for further testing and/or treatment. |
| Thoracic Aortic Aneurysm (TAA) | **Thoracic Aortic Aneurysm (TAA)** is when the aorta (the main blood vessel that comes out of your heart) weakens. The aorta carries blood from your heart to the rest of your body. The upper part of the aorta is called the thoracic aorta. When part of the thoracic aorta balloons out or bulges it is called a **TAA** (thoracic aortic aneurysm). If your aneurysm is over 4 centimeters you may be referred for additional testing. Small aneurysms seen on scans are less worrisome. The testing will help your provider weigh the chances of the aneurysm bursting. A burst causes internal bleeding and this condition becomes very dangerous. Small or slow growing aneurysms are **not** likely to burst and may be watched for a while on further scans. Your provider will discuss your need for further testing and/or treatment. |
