# Supplementary material for: Identification and management of incidental findings in a Veteran’s lung cancer screening program
Source: Respir Res. 2025 Dec 20;27:24. doi: 10.1186/s12931-025-03466-5 (PMC12836813; doi:10.1186/s12931-025-03466-5)
Supplement: Supplementary file 2 — Supplementary Material 2. Additional Table 2. Categories of Incidental Findings Reviewed on LDCT (Colucci_et_al_AddFile2.docx) [file 12931_2025_3466_MOESM2_ESM.docx]

**Additional Table 2. Categories of Incidental Findings Reviewed on LDCT**

| **Pulmonary Findings** | **Non-Pulmonary Findings** |
| --- | --- |
| - Atelectasis | - Abnormalities of Skin or Chest Wall |
| - Azygos Lobe | - Atherosclerosis |
| - Bronchiectasis | - Cardiac Finding (ex. Cardiomegaly, Pericardial Effusion) |
| - Diaphragm-Related Finding | - Cirrhosis |
| - Emphysema | - Coronary Artery Calcifications |
| - Granulomas | - Esophageal Finding or Hiatal Hernia |
| - - Hamartoma | - - Esophageal Thickening |
| - - Interstitial Changes | - - Patulous Esophagus |
| - Multiple Nodules | - Gastroesophageal Reflux Disease (GERD)^a^ |
| - Pleural Effusion | - Gynecomastia |
| - Pleural Plaques | - Miscellaneous Skeletal Abnormalities |
| - Pulmonary Infiltrates | - Bone Spur/Osteophytes |
| - Pulmonary Lymph Nodes | - Degenerative Changes of the Spine |
| - Pulmonary/Vascular (ex. Pulmonary Artery Enlargement) | - Kyphosis |
| - Scarring | - New Fractures |
|  | - - Old or Healed Fractures |
|  | - Other Calcifications |
|  | - Other GI Finding |
|  | - - Adrenal Adenoma |
|  | - - Cholelithiasis |
|  | - - Diverticulosis |
|  | - - Hepatic Steatosis |
|  | - - Liver Cyst |
|  | - - Liver lesions |
|  | - - Spleen Abnormalities |
|  | - Renal |
|  | - Thyroid |
|  | - Vascular Finding (ex. Aneurysm) |
| ^a^Patients were considered to have GERD if they were on a reflux medication or had a diagnosis of GERD in their medical chart | |
