# Supplementary material for: Identification and management of incidental findings in a Veteran’s lung cancer screening program
Source: Respir Res. 2025 Dec 20;27:24. doi: 10.1186/s12931-025-03466-5 (PMC12836813; doi:10.1186/s12931-025-03466-5)
Supplement: Supplementary file 3 — Supplementary Material 3. Additional Table 3. Demographics and Potential Relationship to Total Incidental Findings per LDCT (Colucci_et_al_AddFile3.docx) [file 12931_2025_3466_MOESM3_ESM.docx]

**Additional Table 3. Demographics and Potential Relationship to Total Incidental Findings per LDCT**

|  | **Observations Available** | **Univariable Model**^a^ | **Multivariable Model**^a^**^,^**^b^  **(N = 397)** |
| --- | --- | --- | --- |
| **Continuous Variables – Change in Total Incidental Findings per 10-unit increase** | | | |
| *Age* | 444 | **0.94 (0.59, 1.3)** | **0.97 (0.59, 1.4)** |
| *Pack Years at Consult* | 411 | 0.023 (-0.078, 0.12) | -0.039 (-0.14, 0.064) |
| **Categorical Variables – Difference in Means** | | | |
| *Sex* | 444 |  |  |
| *Female v. Male* |  | -0.40 (-1.4, 0.62) | -0.31 (-1.3, 0.73) |
| *BMI*^c^ | 441 |  |  |
| *Overweight v. Underweight or Normal Weight* |  | -0.21 (-0.95, 0.53) | -0.27 (-0.79, 0.73) |
| *Obese v. Underweight or Normal Weight* |  | 0.088 (-0.64, 0.82) | 0.24 (-0.52, 0.99) |
| *Current Smoker* | 444 |  |  |
| *Yes v. No* |  | -0.46 (-0.93, 0.012) | **-0.52 (-1.00, -0.031)** |
| *Race*^d^ | 431 |  |  |
| *Non-White v. White* |  | -0.47 (-1.2, 0.24) | -0.43 (-1.1, 0.28) |
| ^a^Statistical significance at level *p* = 0.05 represented by bolded font  ^b^Multivariable model adjusting for all variables in the table including Age, BMI, Pack Years Smoked at Earliest LCS Consult, Sex, Current Smoking Status, and Race  ^c^Numeric BMI is categorized based on CDC recommendations for adults: Underweight or Normal Weight (less than 25.0), Overweight (25 to less than 30), and Obese (30 or greater). 8 patients were considered underweight (BMI < 18.5)  ^d^‘Non-White’ race includes: African American or Black (n=48), American Indian or Alaska Native (n=5), Asian (n=2), and Native Hawaiian or Pacific Islander (n=1) | | | |
