# Supplementary material for: Identification and management of incidental findings in a Veteran’s lung cancer screening program
Source: Respir Res. 2025 Dec 20;27:24. doi: 10.1186/s12931-025-03466-5 (PMC12836813; doi:10.1186/s12931-025-03466-5)
Supplement: Supplementary file 4 — Supplementary Material 4. Additional Table 4. Demographics and Potential Associations to Presence of an Actionable Incidental Finding on a LDCT (Colucci_et_al_AddFile4.docx) [file 12931_2025_3466_MOESM4_ESM.docx]

**Additional Table 4. Demographics and Potential Associations to Presence of an Actionable Incidental Finding on a LDCT**

|  |  | **Odds Ratio (95% Confidence Interval)** | |
| --- | --- | --- | --- |
|  | **Observations Available** | **Univariable Model** | **Multivariable Model**^a,b^  **(N = 442)** |
| **Continuous Variables – Presented per 10-Unit Increase** | | | |
| *Age* | 444 | 1.21 (0.690, 2.11) | **1.77 (1.03, 3.05)** |
| *Pack Years at Initial Consult* | 411 | **1.11 (1.00, 1.23)** | 1.11 (0.907, 1.35) |
| **Categorical Variables** |  |  |  |
| *Sex* | 444 |  |  |
| *Female v. Male* |  | 1.13 (0.254, 5.01) | 2.71 (0.815, 8.99) |
| *BMI*^c^ | 441 |  |  |
| *Overweight v. Underweight or Normal Weight* |  | **0.339 (0.131, 0.880)** | **0.410 (0.181, 0.930)** |
| *Obese v. Underweight or Normal Weight* |  | 0.567 (0.249, 1.29) | 0.698 (0.327, 1.49) |
| *Current Smoker* | 444 |  |  |
| *Yes v. No* |  | 0.666 (0.322, 1.37) | 0.902 (0.468, 1.74) |
| *Race*^d^ | 431 |  |  |
| *Non-White v. White* |  | 0.442 (0.103, 1.91) | 0.333 (0.077, 1.45) |
| **^a^**Statistical significance at level *p* = 0.05 represented by bolded font  ^b^Multivariable model adjusting for all variables in the table including Age, BMI, Pack Years Smoked at Earliest LCS Consult, Sex, Current Smoking Status, and Race  ^c^Numeric BMI is categorized based on CDC recommendations for adults: Underweight or Normal Weight (less than 25.0), Overweight (25 to less than 30), and Obese (30 or greater). 8 patients were considered underweight (BMI < 18.5)  ^d^‘Non-White’ race includes: African American or Black (n=48), American Indian or Alaska Native (n=5), Asian (n=2), and Native Hawaiian or Pacific Islander (n=1) | | | |
